# Changes of peripheral lymphocyte subsets and cytokine environment during aging and deteriorating gastrointestinal tract health status

**DOI:** 10.18632/oncotarget.18485

**Published:** 2017-06-16

**Authors:** Jing Wang, Guodong Yang, Dongfang Wang, Kuiliang Liu, Yongchao Ma, Hong Liu, Jing Wu, Min Fang

**Affiliations:** ^1^ CAS Key Laboratory of Pathogenic Microbiology and Immunology, Institute of Microbiology, Chinese Academy of Sciences, Beijing, China; ^2^ University of Chinese Academy of Sciences, Beijing, China; ^3^ Beijing Shijitan Hospital, Capital Medical University, Beijing, China; ^4^ International College, University of Chinese Academy of Sciences, Beijing, China

**Keywords:** aging, gastrointestinal tract disease, NK cells, NKT cells, T cells, Gerotarget

## Abstract

Human immune senescence accompanies with the physical and physiological frailty. The functional change and shift of NK, NKT and T cell subsets by aging have been widely studied. However, it remains largely unclear how the aging and disease conditions affect the distribution of lymphocytes. In the present study, 233 subjects with age range from 20 to 87 year old, including healthy people, people with chronic gastrointestinal tract disease or cancers were investigated. We found that the proportion of NK cells, CD8^+^ T cells and NKT cells remained relatively unchanged with aging. However, NKG2D and CD16 expression level on NK cells decreased with aging indicating impaired NK cell function. Surprisingly, the proportion of NK, NKT and T cells all declined with deteriorating health status from health to chronic gastrointestinal tract disease and cancer. Furthermore, cytokine and chemokine profiles changed with aging, but did not vary with different health status. Our results highlight new evidence for a continuum of change during immunologic aging and show unique data for variations of NK cells, CD8^+^ T cells, NKT cells, and cytokine microenvironment with human aging and health status transformation.

## INTRODUCTION

Immunosenescence is described as a decline in the normal function of the immune system associated with physiologic aging, which leads to increased susceptibility to infection, cancer and autoimmune diseases in aged organisms, including humans [1]. Dysfunction has been defined for both innate immune system providing natural resistance to infections and tumorigenesis, and the adaptive system for acquired and long-preserving immunity. The aging of adaptive immunity has been widely appreciated, because the atrophy of thymus starts with birth and accelerates during adolescence [2]. This modification accompanies with a decrease in the absolute number of T lymphocytes (CD3^+^ T cells), including CD4^+^ T and especially CD8^+^ T subsets [3]. Another important characteristics of immunosenscence is the exhaustion of CD95^-^ virgin T cells which are replaced by the progressive expansion of CD28^-^ T cells among both CD4^+^ and CD8^+^ subsets [4]. CD28^+^ T cells lose the CD28 receptor due to repeated antigenic stimulations and become replicative senescence with a characters of shortened telomeres and reduced proliferative capacity [5].

The innate immune system, which serves as an immunological sentinel defense against microbial pathogens, is also affected at multiple levels during the aging process [6, 7], but the effects and mechanisms remain incompletely understood, particularly in humans [8]. Natural Killer (NK) cells are an important component of innate immunity, playing vital roles in host defense against invading pathogens and malignant transformation [9, 10]. The effect of aging on NK cell function has been extensively studied and often contrasting results emerged [11]. Recent studies have demonstrated that the ratio and number of NK cells are stable or increasing during aging [3, 12]. Nevertheless, the age-related decrease in NK cell activity reported by some investigators might be related to selection criteria, as indicated by the fact that subjects selected following the strict criteria (SENIEUR Protocol) do not present such a diminution [4, 13]. Other researches revealed that lower NK cell activity in elderly subjects is associated with development of infectious morbidity and mortality [14]. Hence, the well-preserved NK cell activity might be an indicator of healthy aging and longevity [3].

Another hallmark of aging is the changing levels of inflammatory cytokines. IL-15 is best known for its effect on the immune system. Significantly elevated serum IL-15 levels were observed in centenarians, suggesting high expression of IL-15 conferred protection from frailty and age-related disease [15]. IL-15 also has important effects on adipose tissue. IL-15 inhibits adipocyte differentiation in culture and obese people have low blood IL-15 levels [16]. Lutz et al posited that increased adipokine and decreased IL-15 levels during aging provide a common mechanism for sarcopenia, obesity, and immune senescence [16]. IL-12, a cytokine capable of directly stimulating NK and T cells to produce IFN-γ, is a central stimulator of Th1 type cytokines. A role for IL-12 in the aging process was indicated in that splenocytes from aged animals cultured in the presence of anti-IL-12 antibodies showed a significant reduction in spontaneous IL-6, IL-10 and IFN-γ production [17]. Thus, IL-12 might be responsible for the dysregulated production of IL-10 and IFN-γ known to occur in aged animals [17]. Recently, “inflamm-aging” has been described, where healthy aging individuals have an active inflammatory status, with high levels of IL-6, IL-1β and tumor necrosis factor-α (TNF-α) [18]. However, how the levels of cytokines change throughout life and the possible effect on immune cell functions in human remain largely unknown.

The relationship between aging and disease is complicated. Aging is a continuum of changes that are related to time and are universal and progressive in everyone [19]. Disease is affected by multiple factors including aging. So far, most immune gerontologic studies focus on subjects strictly selected based on the SENIEUR protocols [20]. In SENIEUR protocol, subjects are selected by strict admission criteria to limit the influence of extrinsic factors such as intercurrent disease and the use of medication. However, the distinction between aging and disease is difficult and theoretical. How the reshaping of immune cells and cytokine levels in inflammation or cancer subjects with aging are unknown. In the current study, we studied the changes of peripheral lymphocyte subsets and cytokine profiles of 233 subjects ranging from 20 to 87 years old, including healthy people, people with chronic gastrointestinal tract disease or cancers. This is the first report, to our knowledge, that evaluates the changes of immune status with multivariates (age and health status) by the General Linear Model (GLM) analysis.

## RESULTS

### The group information of the 233 studied subjects

**Figure 1:**
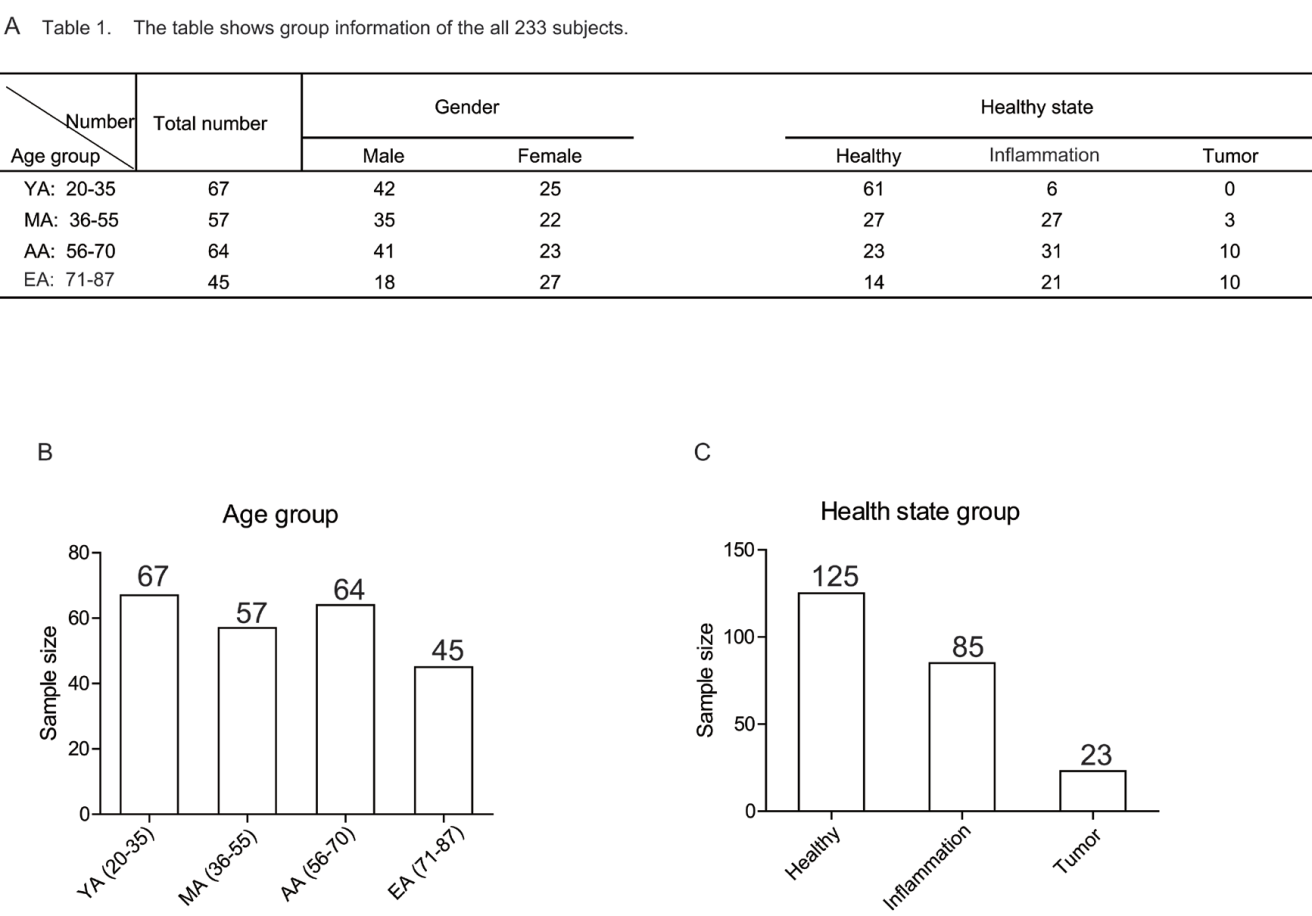
The group information of 233 studied subjects **A.** Table 1 shows detailed group information of all the subjects. Numbers represent the sample size in the indicated group. **B.** The scheme of the different age groups. The numbers in the bar graph indicate the size of subjects in the indicated age group. **C.**The scheme of the different health status groups. The numbers in the bar graph indicate the size of subjects in the indicated health status group.

A total of 233 subjects aged from 20 to 87 were studied. Of all these subjects, the basic information (such as age, sex, health status) was collected. The subjects were divided into 4 groups based on age: young adults (YA, ages 20-35, 67 subjects), middle aged adults (MA, ages 36-55, 57 subjects ), aging adults (AA, ages 56-70, 64 subjects) and elderly adults (EA, ages 71-87, 45 subjects) (Figure 1B). Among all the subjects, 125 were healthy subjects (healthy group), 85 subjects had chronic gastrointestinal tract diseases (inflammation group), and 23 subject had gastrointestinal tract cancers (tumor group) (Figure 1C).

The process of senescence has been studied since 1961 [12]. The normal process of senescence is affected by many factors, such as infection history, lifestyles and stress. For decades, studies in the normal senescence have followed on the criteria proposed by the SENIEUR (from SENIorEURopean) protocol, which provided strict criteria for the individuals chosen for study [21]. However, it is generally unclear how other factors, such as healthy condition, influence the aging process. In this study, we investigated the changes of lymphocyte subsets and cytokines with aging without eliminating the health status, which includes healthy individuals (healthy group) and individuals with chronic gastrointestinal tract diseases (inflammation group) or gastrointestinal tract cancer (tumor group). The detailed information of the samples and groups was listed in Figure 1A.

### Gating strategy and functional analysis of different lymphocytes

The changes of the percentage and phenotype of NK, T and NKT cells with aging have been extensively studied in the past [3, 11]. However, due to the sample size and selecting strategy, there might have some limitations in the gerontology study.

**Figure 2:**
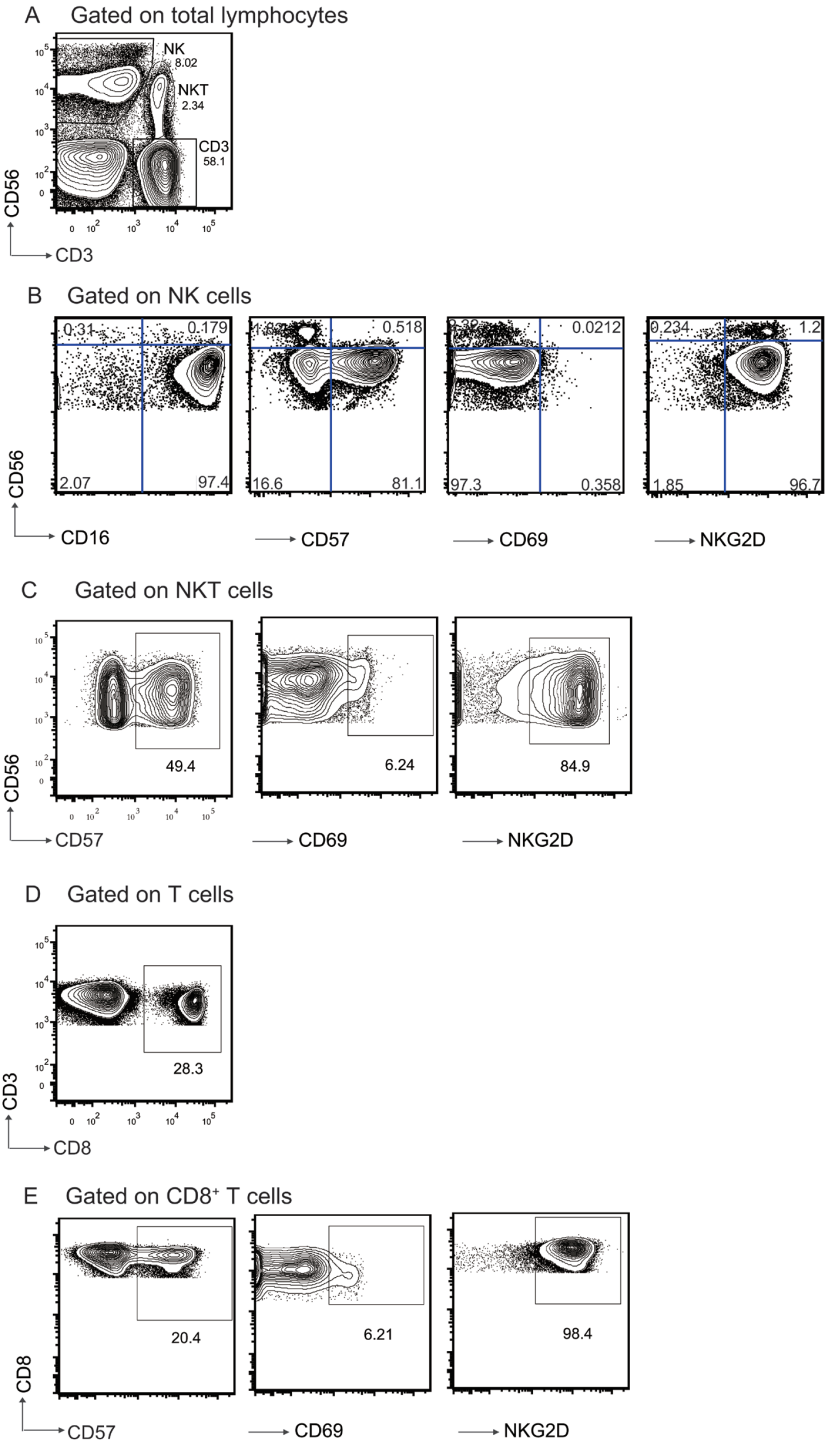
Gating strategy and representative flow cytometric analysis of lymphocytes populations form the subjects. A Identification of different cell populations in PBL based on CD3 and CD56 expression. NK cells(CD3^-^CD56^+^); classical T cells (CD3^+^CD56^-^); NKT cells (CD3^+^CD56^+^). Frequency of each cell population is shown beside the population. **B.** Gated on CD3^-^CD56^+^ NK cells. CD16, CD57, CD69, and NKG2D expression level on NK cells were shown. **C.** Gated on CD3^+^CD56^+^ NKT cells. CD57, CD69, and NKG2D expression on NKT cells were shown. **D.** CD8 expression on CD3^+^CD56^-^ T cells. **E.** Gated on CD8^+^ T cells. CD57, CD69, and NKG2D expression on CD8^+^ T cells were shown. Numbers represent the proportions of the indicated molecules in the gated cell populations.

The cell populations were gated by the strategies shown in Figure 2. Gate strategy (Figure 2A) showed the staining of cell populations of interest in lymphocyte gate based on CD3 and CD56 expression. Figure 2B showed the analysis for gating on the CD56^+^CD3^-^ NK cells, the expression level of CD16, CD57, CD69 and NKG2D on NK cells. Figure 2C showed the analysis for gating on CD56^+^CD3^+^ NKT cells, the expression level of CD57, CD69 and NKG2D on NKT cells. Among the CD3^+^ T cells, we further analyzed on CD8^+^ T cells (Figure 2D), we determined the expression level of CD57, CD69 and NKG2D on CD8^+^ T cells (Figure 2E).

The General Linear Model (GLM) is used for the analysis of age, health status, age and healthy mutual related changes in T, NK, NKT cell population. The interaction of age and health status does not significantly affect the cell population and cytokine level in peripheral blood (F test *p* value > 0.05, data not shown).

### NK cell percentage remains relatively unchanged, but NKG2D and CD16 expression level on NK cells decrease with aging

**Figure 3:**
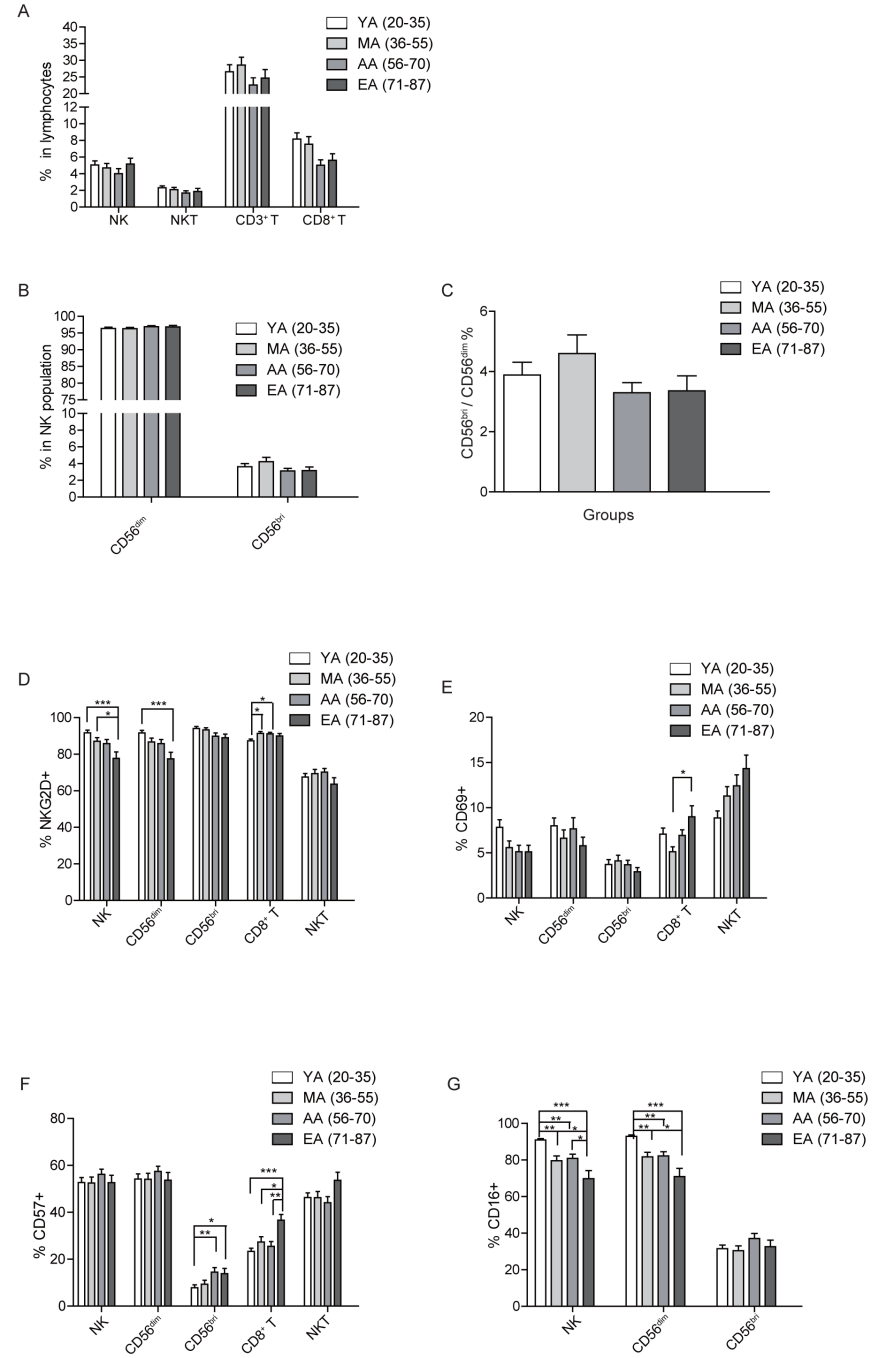
Age related changes in surface receptor expression. A Percentages of NK, NKT, CD3^+^, and CD8^+^ T cells in PBL are shown as a mean ± SEM based on different age group. **B.** The age related percentage change of CD56^dim^ and CD56^bri^ NK subsets in NK cells shown as a mean ± SEM. **C.** Distribution of CD56^bri^: CD56^dim^ ratio in different age groups. **D.**-**G.** The expression level of NKG2D(D), CD69 **E.**, CD57 **F.**, and CD16 **G.** on the indicated cell populations. Each graph is shown as a mean (±SEM). *p < 0.05, **p < 0.01,***p < 0.001 by Turkey HSD test comparison of the four age groups post hoc analyses.

Investigations of subjects selected following SENIEUR Protocol showed a preserved NK activity with aging. However, age-related changes in subjects selected not strictly based on the protocol were also indicated in some other reports [4, 13]. By phenotypic analysis of peripheral blood lymphocytes (PBL) from all subjects studied, we found that the NK cell percentage did not change significantly with aging (Figure 3A). However, there was a slight decrease of NK cell percent in the AA group (ages 56-70) compared to YA (ages 20-35), MA (ages 36-55) and EA groups (ages 71-87). Although did not reach statistic significance, NK cell percent slightly increased in the EA group (ages 71-87). Our results differ with the gradual increasing trend of NK cell percentage with aging as shown by previous reports [1, 3]. One possible reason might be the different grouping strategies and sample sizes. In an previous study, the total 73 healthy subjects aging from 5 to 77 year old were divided into 3 groups according to age: children (ages < 18, 15 subjects), adults (ages 19-59, 37 subjects), and elderly adults (ages > 60, 21 subjects) [1]. In our study, we divided samples (totally 233 subjects aging from 20 to 87) into 4 groups: young adults (YA, ages 20-35, 67 subjects), middle aged adults (MA, ages 36-55, 57 subjects ), aging adults (AA, ages 56-70, 64 subjects) and elderly adults (EA, ages 71-87, 45 subjects). With more subjects in each group and a more elaborate age division, our data revealed the subtle changes of NK cell population with aging.

Human NK cells can be divided into two subsets based on the expression level of CD56 and CD16. CD56^dim^CD16^+^ NK cells have high cytotoxic function and CD56^bri^CD16^+/-^ NK cells have immune regulation function by producing cytokines [22]. Previous studies shown that the NK cell subpopulations are affected by aging [1, 23]. By gating strategy in Figure 2, we further identified the change of CD56^bri^, CD56^dim^ and their ratio with aging. In accordance with the trend in total NK cell population, there was no significant age related change in CD56^dim^, CD56^bri^ NK cells or ratio of CD56^bri^ to CD56^dim^ (Figure 3B and 3C). Even though no statistic differences were observed, the percentage of CD56^dim^ NK cells had a trend of increasing with aging (Figure 3B), which is in accordance with a previous report indicating that the percentage of CD56^dim^ NK increases in the elder compared to the very young group [1].

Our results showed that the percentage of NK cells, especially the mature CD56^dim^ subset, were kept at a higher level in the elder individuals (Figure 3A and 3B). To further investigate changes of NK cell phenotype, we next measured the surface expression level of NKG2D, CD69, CD16 and CD57 on total NK cells, CD56^dim^ and CD56^bri^ NK cell subsets. The expression level of activating receptor NKG2D was significantly decreased in total NK cells (F test *p* = 0.004) and CD56^dim^ NK cell subsets (F test *p* = 0.003) with aging. Importantly, the NKG2D expression level showed an gradually decreased trend with aging, the level in EA group (ages 71-87) was significantly lower compared to that of the YA (ages 20-35) and MA group (ages 36-55)(Figure 3D).

CD69 is an early activation marker in NK and T cells [24]. Analysis of the CD69 expression on total NK, CD56^dim^, and CD56^bri^ NK cells showed that CD69 expression level was slightly decreased with aging, but did not reach statistical significance (Figure 3E). The CD57 is a marker of terminal differentiation on human CD8^+^ T cells [25]. Previous study indicated that CD57^+^ NK cells were highly mature and might be terminally differentiated, proliferated less when stimulated with target cells and/or cytokines, and the frequency of CD57^+^ NK cells increased with aging [26]. Surprisingly, we found that the CD57 expression level on total NK or CD56^dim^ NK cells remained unchanged from YA (ages 20-35) to EA (ages 71-87). However, there was a significant increase of CD57 expression on CD56^bri^ NK cells (F test *p* = 0.001) in the EA (ages 71-87) and AA group (ages 56-70) compared to that of YA group (ages 20-35) (Figure 3F).

CD16 is an Fc receptor which mediates the antibody-dependent cell-mediated cytotoxicity (ADCC) and is highly expressed on CD56^dim^ NK cells [27]. CD16 expression among total NK (F test *p* = 0.032) and CD56^dim^ NK cells (F test *p* = 0.027) was significantly decreased with aging (Figure 3G). According to the HSD post hoc analyses, CD16 expression on total NK and CD56^dim^ NK cells had statistic significant differences among each age group except for the MA (ages 36-55) and AA group (ages 56-70)(Figure 3G). There was no significant differences of CD16 expression level in the CD56^bri^ NK cells between different age groups. Our results were different from previous studies, which showed that CD16 expression was not affected by aging [12, 28].

Above all, the expression level of NKG2D, CD16 on NK cells decreased with aging, which indicate phenotypic changes of NK cells with aging. Due to the limited amount of PBL, we did not determine the NK cell cytolytic functions in our current study.

### Expression level of CD69 and CD57 is increased in CD8^+^ T cells with aging

Previous stuides showed a decrease in the absolute number of T lymphocytes (CD3^+^), including CD4^+^ T and especially CD8^+^ T subsets, with aging [3]. However, we found that the percentage of CD3^+^ T cells remained relatively stable with aging. The percentage of CD3^+^ T cells reached peak in the MA group (ages 36-55), while stayed low in the AA group (ages 56-70). Nevertheless, there was no significant differences among all the different age groups. Among the T cells, CD8^+^ T cells showed a slightly decreasing trend in the AA (ages 56-70) and EA group (ages 71-87) compared to the YA (ages 20-35) and MA group (ages 36-55). Again, no statistical significances were reached among all the groups (Figure 3A).

To further assess the effect of aging on CD8^+^ T cell repertoire, we checked the expression of NKG2D, CD69 and CD57 in each age group. NKG2D expression level significantly increased in the MA (ages 36-55) and AA group (ages 56-70) compared to that of YA group (ages 20-35)(Figure 3D). The expression level of CD69 showed a slight decrease in the MA group (ages 36-55) compared to YA group (ages 20-35). However, from MA (ages 36-55) to AA (ages 56-70) and EA group (ages 71-87), CD69 expression showed a gradually increasing trend, the expression of CD69 (F test *p* = 0.029) on EA group (ages 71-87) was significantly increased compared to that on MA group (ages 36-55) (Figure 3E). As a marker of terminal differentiation of human CD8^+^ T cells, the expression of CD57 on CD8^+^ T cells (F test *p* = 0.000) showed a significant increase in the EA group (ages 71-87) compared to all the other 3 age groups (Figure 3F), which is consistent with previous report [29].

### NKT cells remain largely unchanged with aging

Natural killer T (NKT) cells are a heterogeneous group of T cells that share properties of both T cells and natural killer cells [30]. The NKT cells recognize lipid antigens and play both effector and regulatory roles in infectious, tumor and autoimmune diseases [30]. The cell numbers or NKG2D expression on NKT cells were reported to remain largely unchanged with aging [1]. Consistent with previous studies, we found that NKT cell percentage remained stable with aging(Figure 3A). Furthermore, there was no significant differences of NKG2D expression between the four different age groups, albeit that in the EA group (ages 71-87), the NKG2D expression level was lower compared to other three age groups (Figure 3D). Even though all the groups did not reach any statistical significance, the expression of CD69 on NKT cells gradually increased with aging (Figure 3E). Moreover, the expression of CD57 on NKT cells also slightly increased in EA group (ages 71-87) (Figure 3F). Thus, NKT cells remained largely stable with aging, while the expression of CD69 and CD57 on NKT cells showed a trend of increase with aging.

### NK, NKT and T cells decline with deteriorating health status

NK and CD8^+^ T cells (cytolytic T lymphocytes, CTL) play indispensable roles in anti-tumor immune response. NK and CD8^+^ T cells mainly use two major pathways to eliminate tumor cells: the perforin/granzyme-containing granule exocytosis pathway, or the death-receptor-ligand pathway [31]. Previous studies on the changing of immune cell populations with aging mostly followed the strict standard SENIEUR Protocol [20, 32]. To evaluate the effects of aging and health conditions on immune cell populations, we further analyzed the influence of digestive tract diseases on immune cell populations following the renewed SENIEUR Protocol concentrating on the interaction of aging and disease [20].

**Figure 4:**
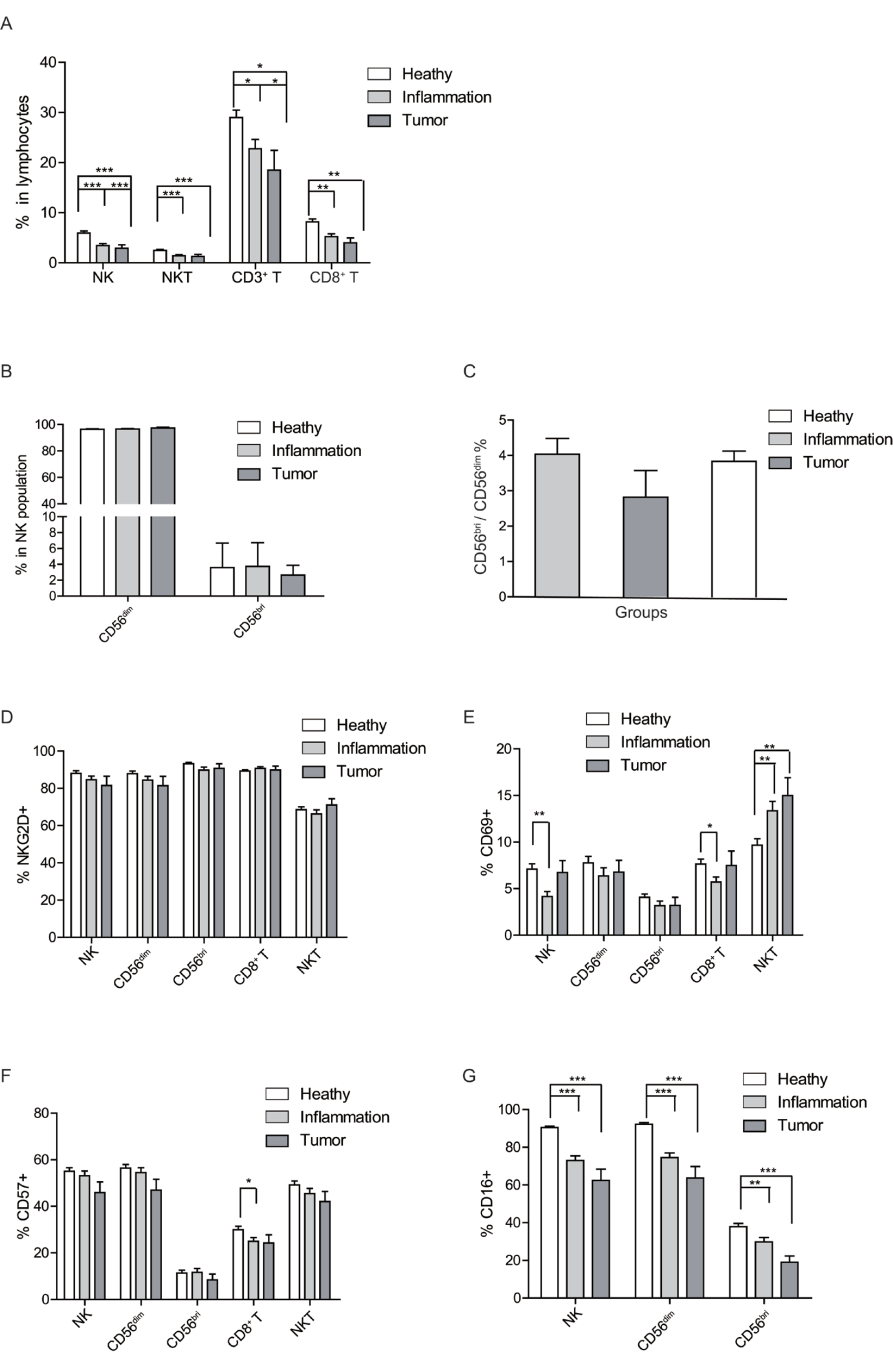
Health status-related changes of lymphocyte population and surface markers. A Percentages of NK, NKT and CD3^+^, CD8^+^ T cells in peripheral lymphocytes are shown as a mean (±SEM) based on different health status group. **B.** The health status related percentage change of CD56^dim^ and CD56^bri^ in NK cells shown as a mean (±SEM). **C.** Distribution of CD56^bri^: CD56^dim^ ratio in different health status groups. **D.**-**G.** The expression level of the labeled surface markers on the indicated cell populations, **D.** NKG2D, **E.** CD69, **F.** CD57, and **G.** CD16. Each graph is shown as a mean (±SEM). *p < 0.05, **p < 0.01,***p < 0.001 by Turkey HSD test comparison of the three health status groups post hoc analyses.

As showed in Figure 4A, the percentages of NK cells (F test *p* = 0.000), NKT cells (F test *p* = 0.000), CD3^+^ T cells (F test *p* = 0.004), and CD8^+^ T cells (F test *p* = 0.006) in the lymphocytes declined with the health status changing from healthy, inflammation to tumor. The subpopulation of NK cells was not significantly affected by the different health status (Figure 4B), neither was the ratio of CD56^bri^ to CD56^dim^ (Figure 4C). Therefore, total NK cells declined with deteriorating health status whereas the ratio of subpopulations were not changed.

Further, NKG2D expression level remained constant on NK, CD8^+^ T and NKT cells among the healthy, inflammation and tumor groups (Figure 4D). However, CD69 expression on total NK cells (F test *p* = 0.042) and CD8^+^ T cells (F test *p* = 0.012) was significantly decreased in the inflammation group compared to the healthy group, while no statistical significance was reached in the CD56^bri^ or CD56^dim^ NK cells subsets (Figure 4E). Interestingly, the CD69 expression on NKT cells gradually increased with the deteriorating health status. There was a significant increase of CD69 expression on NKT cells in the inflammation group and tumor group compared to that of the healthy group (Figure 4E). There was no significant change of CD57 expression on NK and NKT cells among the three different health groups. However, compared to healthy group, the expression of CD57 on CD8^+^ T cells (F test *p* = 0.000) was significantly decreased in the inflammation group (Figure 4F). Further, CD16 expression presented gradually decreasing trends on total NK (F test *p* = 0.000), CD56^dim^ NK (F test *p* = 0.027), and CD56^bri^ NK cells (F test *p* = 0.000) from healthy group to inflammation group, and then to tumor group (Figure 4G).

In summary, the frequencies of NK, NKT and T cells all decreased with deteriorating health status. More importantly, we found that the CD16 expression level showed a gradually decreasing trend between the subjects with deteriorating health status, which indicates that NK cell functions, especially cytolytic function might become impaired in the inflammation and tumor groups. Our data also displayed a phenomenon that the immune status of inflammation might be in a middle condition between health state and tumor state, which further infers the interim between inflammation and tumor/cancer [33].

### Age-related changes of cytokines and chemokines

**Figure 5:**
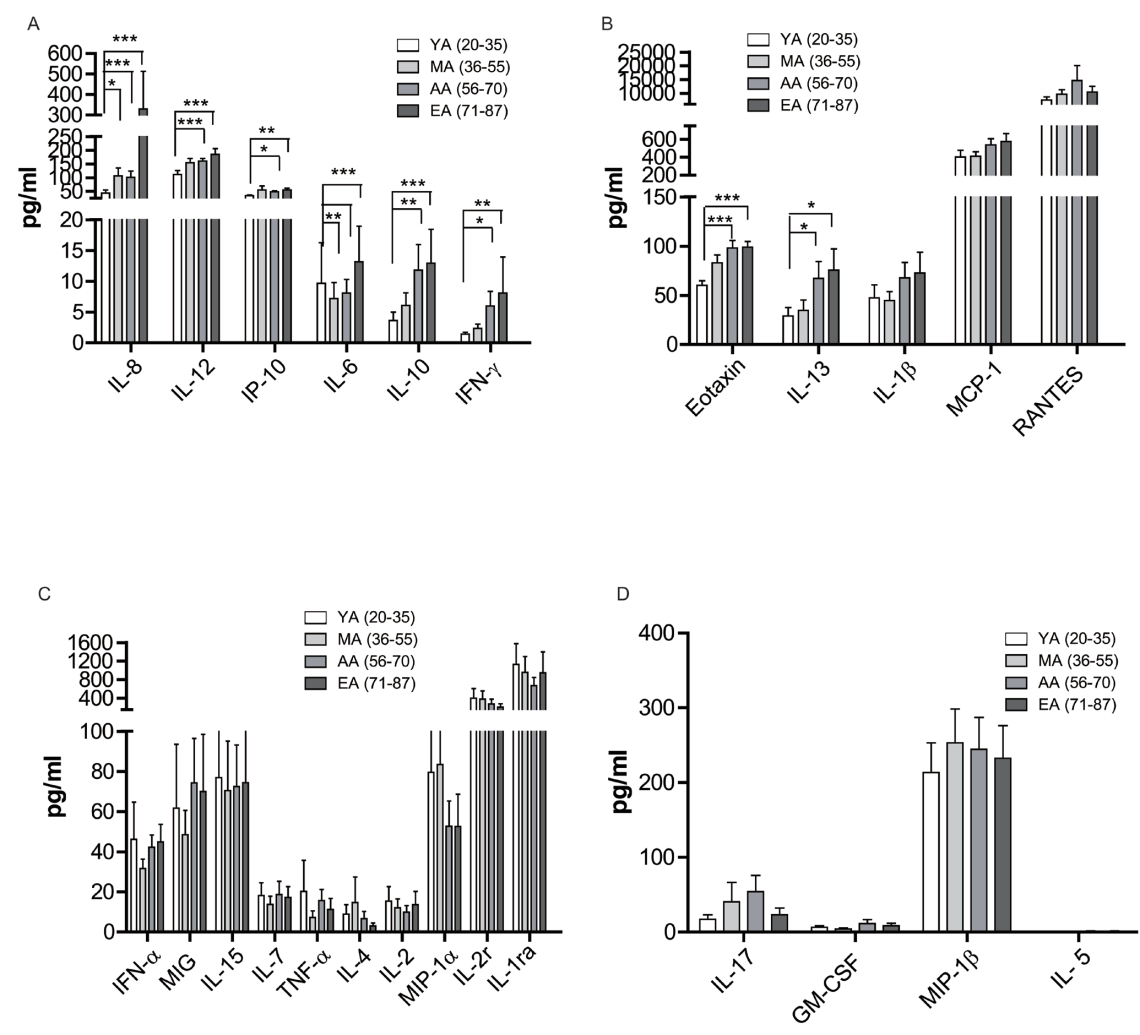
Age related changes in cytokine levels in peripheral blood The peripheral blood were centrifuged and serum were collected. The cytokine level was measured by a Luminex assay. **A.**-**D.** Individual cytokine level in four age groups. Data is shown as a mean (±SEM). *p < 0.05, **p < 0.01,***p < 0.001 by Turkey HSD test comparison of the four age groups post hoc analyses.

Previous studies indicated that healthy aged individuals have a reduced capacity to produce IFN-γ and IL-2 (as the main T-helper-1 (Th1) cytokines) [34]. However the Th2 cytokines, such as IL-4 and IL-10, are produced at higher levels when compared to stimulated lymphocytes from young donors [34, 35]. These results indicate a dysregulated Th1/Th2 system predominated by Th2 functions in the aged individuals [34, 35]. To further investigate the effect of aging on the host cytokine environment in general, we detected the concentration of 25 cytokines in the peripheral blood of all the four age groups. As shown in Figure 5A and 5B, IL-8 (F test *p* = 0.001), IL-12 (F test *p* = 0.000), IP-10 (F test *p* = 0.000), IL-6 (F test *p* = 0.002), IL-10 (F test *p* = 0.001), IFN-γ (F test *p* = 0.002), Eotaxin (F test *p* = 0.000) and IL-13 (F test *p* = 0.001) showed a remarkable increase in the AA (ages 56-70) and EA group (ages 71-87) compared to the YA group (ages 20-35).

Meanwhile IL-1β, MCP-1 and RANTES also showed a trend of slightly increasing with aging, but did not reach statistical significance. On the other hand, IL-4, MIP-1α, and IL-2r showed a slight decrease in the EA group (ages 71-87) compared to YA (ages 20-35). Other cytokines, such as IFN-α, MIG, IL-15, IL-7, TNF-α, IL-2, IL-17, GM-CSF, MIP-1β, and IL-5 did not show obvious differences between the four age groups (Figure 5C and 5D). In summary, our data revealed the general changes of cytokines with aging, the mechanisms of how cytokine shift affect the function of immune cells and their impact to age-related diseases require further in-depth investigations.

### Cytokine micro-environment remains largely unchanged with different health status

Many researches linking cancer and inflammation have indicated that individuals suffering from chronic inflammation are more prone to developing cancer [33]. The etiology of cancer is complex that involves both genetic and environmental factors [7]. The roles of IL-6, GM-CSF, IFN-γ in the link of inflammation and cancer have been indicated before [36, 37]. Briefly, IL-6 down regulates p53 expression and activity by stimulating ribosome biogenesis, connecting inflammation to cancer [36]. And the deficiencies in GM-CSF and IFN-γ promote the transition of inflammation to cancer [37]. However, the changes of cytokines with different health status remain largely unexplored. Therefore, we next investigated possible health status-related changes in the host environment.

**Figure 6:**
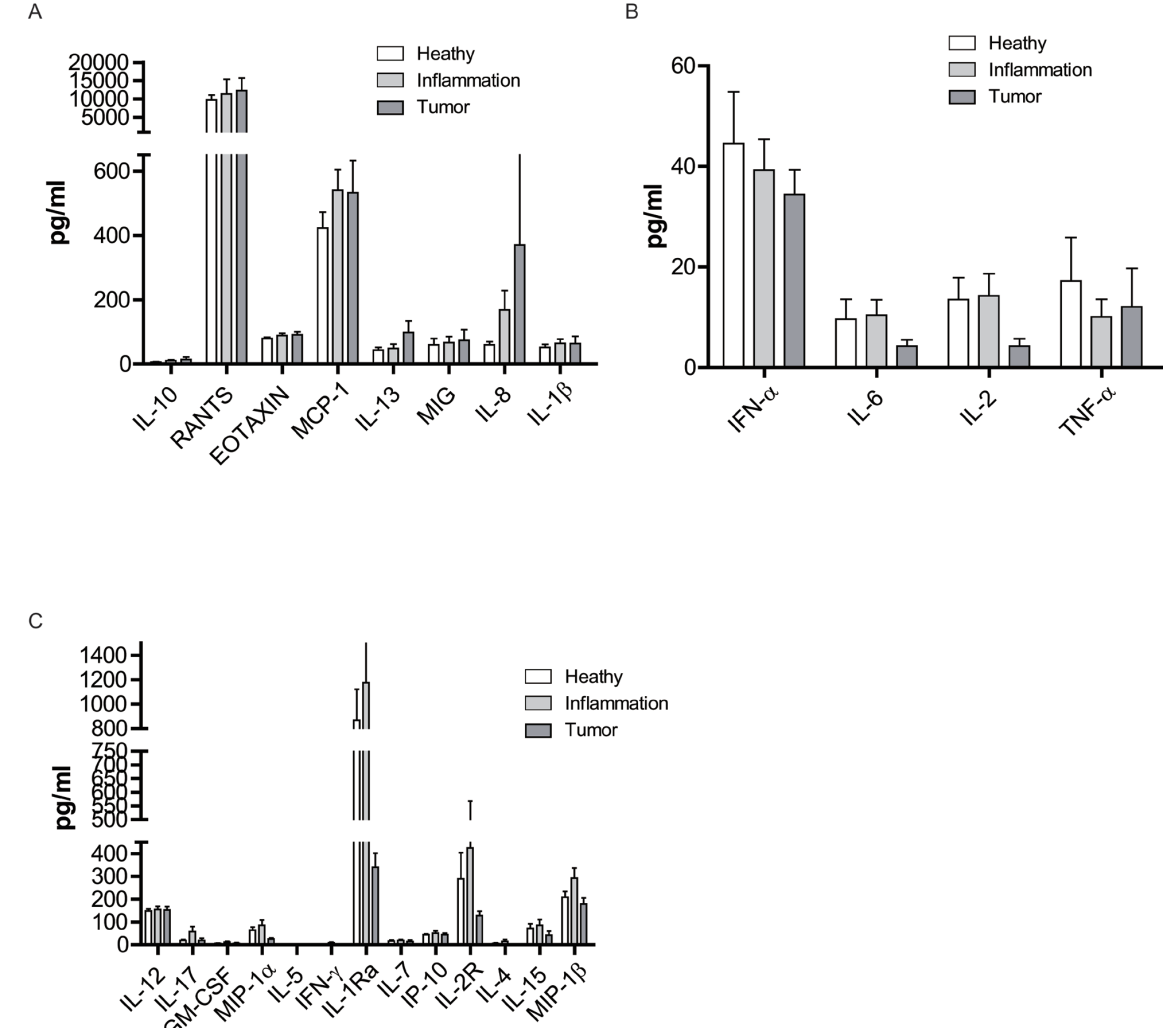
Health status-related changes in cytokine levels in peripheral blood The cytokine and chemokine levels in the serum of all the subjects were measured by a Luminex assay. **A.**-**C.** Individual cytokine level in three health status groups. Data was shown as a mean (±SEM). *p < 0.05, **p < 0.01,***p < 0.001 by Turkey HSD test comparison of the three health status groups post hoc analyses.

Even though there were no significant changes of cytokines with different health status, the cytokines were divided into 3 groups according to the trend with the deteriorative health status. (1) IL-10, RANTES, Eotaxin, MCP-1, IL-13, MIG, IL-8, and IL-1β presented a progressively increasing trend in the peripheral blood (Figure 6A); (2) IFN-α IL-6, and IL-2 declined from healthy to inflammation or tumor, while TNF-α showed a decreased trend in the inflammation group (Figure 6B); (3) IL-12, IL-17, GM-CSF, MIP-1α, IL-5, IFN-γ, IL-1r, IL-7, IP-10, IL-2r, IL-4, IL-15, and MIP-1β showed a slight increase in the inflammatory group (Figure 6C). Therefore, the health status did not significantly affect the cytokine micro-environments. However, different cytokines displayed distinct patterns with varied health status which might indicate the subtle changes of cytokine micro-environments in the transition from health to diseases (inflammation or cancer).

## DISCUSSION

Because of the improvements in living conditions and medical care, the current increase of aged population will present great challenges in 21^st^ century and promote the research in immunesenescence. The immune functions of the elderly are infected by many factors, such as living environments, their health and mental status. To exclude the influence of extrinsic factors, the SENIEUR protocol (from SENIorEURopean) [21] were designed, which is a strict admission criteria of selecting subjects to ensure healthy aging. However, the relationship between aging and diseases are complicated and not exclusive in many aspects. Thus, the SENIEUR protocol was modified and improved with the aim of reviving the interaction between aging and disease on immune system [20]. In the present study, besides age, the health status of gastrointestinal tract was considered as another variable. We used the GLM (General Linear Model) to analyze the effect of two variables, age and health status on the changes of lymphocyte phenotype and cytokine microenvironment.

From 233 individuals aged from 20 to 87 years old, we found that the percentages of NK, CD8^+^ T and NKT cells were relatively sustained with aging. Previous reported decline in NK cell reactivity might be related with changes in the expression of inhibitory and activation receptors [38]. Although NK cell percent remained largely unchanged with aging, however, we found that the expression levels of NKG2D and CD16 were significantly decreased with aging on total NK cells or CD56^dim^ NK cells, indicating the possible decline of NK cell cytolytic functions with aging. Previous studies showed that neither CD16 expression nor CD16 function was altered in the elderly [38]. One possible reason for the differences might be the large sample size and more elaborate group strategy in our study. Similar to previous studies [1], we also found that the percent of NKT cells did not show significant changes with aging. However, even did not reach statistical difference, the expression level of early activation marker CD69 on the NKT cells showed an gradual increase with aging, which might indicate that activated NKT cells accumulate in blood as people age.

Aging of the adaptive immune system is widely appreciated because of the atrophy of thymus [39]. Indeed, T cells in the aged show decreased ability to promote B cell activation and differentiation [40], suppressed proliferative response to mitogens and antigens [41], and reduced ability to generate allospecific CTLs [39]. Consistent with a previous report [3], we found that the percentage of CD3^+^ and CD8^+^ T cells displayed a slight decrease with age. The level of NKG2D expression on CD8^+^ T cells remained high with aging, and kept at higher levels in MA (ages 36-55) and AA groups (ages 56-70). However, the CD8^+^ T cells from EA group (ages 71-87) expressed highest level of the earlier activation marker CD69 and the terminal differentiation marker CD57 among the four aging groups, which indicates the accumulation of activated and terminally differentiated CD8^+^ T cells with aging.

NK cells protect host against malignancies and the antitumor functions of NK cells are strictly regulated [42]. Previous studies indicated that as early as the localized primary breast tumor stage, the NK presented a strong drop in IFN-γ production in response to either Type-I IFN or TLR7/8 ligand [43]. In the present study, we find that NK cell percentage in total lymphocytes presented a gradual decrease during the changing from healthy to cancer (gastrointestinal tract). Furthermore, total NK cells or CD56^dim^, CD56^bri^ NK subsets gradually decreased the CD16 expression from healthy state to cancer. We did not perform the cytotoxicity assay due to the limited lymphocytes from PBL. CD16 is an Fc receptor which mediates the antibody-dependent cell-mediated cytotoxicity (ADCC) [27]. The decreased CD16 expression on total NK, CD56^dim^, CD56^bri^ NK cells might reveal the decreased ADCC effector function with the deteriorative health status to some extent. Our results might provide a new cue for the immune therapy based on natural killer cells. The immune therapies in the last decades are mostly based on the clinical successes of immune checkpoint blockade and adoptive T cell transfer, which motivates our own immune system to fight against the cancer cells [44, 45]. Although NK cells play roles in the host defense of tumor, the immune therapy based on NK cells is limited by the requirement of a large number of functional NK cells [46]. Based our findings, a new strategy which restore the expression of CD16 on NK cells should be taken into consideration, which might guarantee the function of autologous NK cells.

The adaptive immune system suppression in individuals with tumors has been previously reported [43]. Herein, we found that the CD3^+^ T cells and CD8^+^ T cells showed a gradual decrease from health changing to cancer. Further, NKT cells also decreased with deteriorative health status. Moreover, CD69 expression on NKT cell gradually increased with deteriorative health status, indicating NKT cells might become activated with deteriorative healthy conditions. These results showed the dynamical changing of lymphocytes from healthy to cancer in the peripheral blood [47].

The relationship between inflammation and tumor or cancer has been widely appreciated [33]. However, the cytokine profiles of the healthy, inflammation and tumor had not been extensively studied. In the current study, we examined the profiles of 25 cytokines and chemokines in the serum of individuals from healthy group, inflammation group and tumor group. Briefly, IL-12, IL-17, GM-CSF, MIP-1α, IL-5, IFN-γ, IL-1Rα, IL-7, IP-10, IL-2R, IL-4,IL-15,MIP-1β levels were higher in the inflammation group and kept relative lower level in healthy and tumor groups. This might provide new insights into the cytokines which might take parts in the transition to tumor or cancer. But the mechanisms of how these cytokines function in the transition remain obscure.

Previous studies revealed that IL-12 induce NK cells and T cells to produce IFN-γ, it also enhances the cytotoxicity of NK cells and CTL [48, 49]. Burly argued that EOTAXIN (CCL11) can selectively activate eosinophil and its increasing level with aging in serum has been reported before in both human and mice [6]. The increasing concentration of IL-12 and EOTAXIN with aging reveals the possibility of these two cytokines play important roles in the elder immune system.

In the process of data analysis, we reserved two variables, age and health status, respectively. The interaction of both variables on the peripheral lymphocytes and cytokines were also examined. Consequently, the interaction of age and health status has no significant effect on the distribution of subpopulation of NK, T, NKT cell and cytokine levels, which might provide precondition that we can investigate immune senescence in individuals with less strict selection standard.

In summary, our findings revealed the age- and health-related changes in peripheral lymphocytes and cytokine environment. The GLM model takes more factors which might have effects on the variables into consideration. We have demonstrated several age and health status related changes in the expression of receptors in different lymphocyte populations. Surprisingly, we found the cytotoxic activity of NK cells might decrease with aging. Further, we found the gradual decrease of NK, NKT and T cells from healthy to cancer group. We also observed some cytokine level changes associated with aging and health status. The factors which promotes the transition from inflammation to tumor still need to be further elucidated.

## MATERIALS AND METHODS

### Subjects

This study was performed with the informed consent of the donors. The experimental design and protocols used in this study were approved by the Regulation of the Institute of Microbiology, Chinese Academy of Sciences of Research Ethics Committee (Permit Number: APIMCAS2017029). A total of 233 subjects aged from 20 to 87 were studied.

### Serum collection and PBMC isolation

3-5 ml peripheral blood was collected from the subjects. The peripheral blood were centrifuged at 672 g for 4min at room temperature. The serum were collected and stored at -80 ^o^C. The cell pellets were washed once with D-Hanks buffer, then the human peripheral blood mononu*c*lear *c*ells ( PBMC) were isolated with PBMC separation medium (TBD science, LTS1007) according to the manufacturer’s instruction. The cells were washed twice with complete RPMI-1640 medium.

### Flow cytometry

The phonotypical analysis of peripheral blood lymphocytes (PBL) was performed by staining PBMC with surface molecules. The following mAb and staining reagents were used: PE conjugated anti-human CD56 mAb (Clone: B-A19), Percp conjugated anti-human CD3mAb (Clone:OKT-3), APC conjugated anti-human CD16 mAb (Clone:H116a), FITC conjugated anti-human CD57 mAb (Clone:HCD57) were purchased from Sungene Biotech (Tianjin, China); Pacific blue conjugated anti-human CD8 mAb (Clone:RPA-78), APC-cy7 conjugated anti-human CD69 mAb (Clone:FN50), PE-cy7 conjugated anti-human NKG2D mAb (Clone:1D11) were purchased from Biolegend. The stained cells were analyzed by flow cytometry using LSRFortessa (BD Biosciences). Data were analyzed with Flowjo software (Treestar Inc.).

### Cytokine analysis

The cytokine levels in the serum were measured using the Human Cytokine Magnetic 25-Plex Panel kit (LHC009, Invitrogen) according to the manufacturer’s instruction and read on Luminex 100 (BioRad).

### Statistical analyses

All data were analyzed by IBM SPSS statistics 19. The General Linear Model(GLM) was used for multivariate analysis of variance. The Turkey HSDpost hoc analyses were performed when the *p* values by F test is < 0.05. Significant differences between different age and health status data were identified by Turkey HSDpost hoc analyses for each group. A *p* value < 0.05 was considered sufficient to reject the null hypothesis. The graphs were made with GraphPad Prism 5 (GraphPad Software, San Diego, CA).
